# The impact of variable commitment in the Naming Game on consensus formation

**DOI:** 10.1038/srep41750

**Published:** 2017-02-02

**Authors:** Xiang Niu, Casey Doyle, Gyorgy Korniss, Boleslaw K. Szymanski

**Affiliations:** 1 Rensselaer Polytechnic Institute, Social Cognitive Networks Academic Research Center, Troy, NY, 12180, USA; 2 Rensselaer Polytechnic Institute, Department of Computer Science, Troy, NY, 12180, USA; 3 Rensselaer Polytechnic Institute, Department of Physics, Applied Physics, and Astronomy, Troy, NY, 12180, USA

## Abstract

The Naming Game has proven to be an important model of opinion dynamics in complex networks. It is significantly enriched by the introduction of nodes committed to a single opinion. The resulting model is still simple but captures core concepts of opinion dynamics in networks. This model limitation is rigid commitment which never changes. Here we study the effect that making commitment variable has on the dynamics of the system. Committed nodes are assigned a *commitment strength, w*, defining their willingness to lose (in waning), gain (for increasing) or both (in variable) commitment to an opinion. Such model has committed nodes that can stick to a single opinion for some time without losing their flexibility to change it in the long run. The traditional Naming Game corresponds to setting *w* at infinity. A change in commitment strength impacts the critical fraction of population necessary for a minority consensus. Increasing *w* lowers critical fraction for waning commitment but increases this fraction for increasing commitment. Further, we show that if different nodes have different values of *w*, higher standard deviation of *w* increases the critical fraction for waning commitment and decrease this fraction for increasing commitment.

Attempts to understand social interactions on networks has led to many diverse models, each balancing the needs for capturing the essence of network dynamics while keeping the model simple and efficiently computable. One emergent model that effectively captures the large scale effects of idea spreading using only simple pairwise interactions between nodes is the binary agreement (BA) model, a two word version of the naming game model^1^,^2^,^3^,^4^,^5^,^6^,^7^,^8^. In this model, each node in a network is holding one or both of two possible opinions. Each time step speaker and listener are chosen randomly the first from the network, and the second from the nearest neighborhood of the speaker. Then, the speaker chooses an opinion from its list to share with the listener. If the listener already holds this opinion, then both the speaker and listener keep only that opinion. Otherwise, the listener simply adds the new opinion to its list. Allowed to evolve this way, the system will eventually reach a stable consensus state in which every node holds the same opinion.

One of the main benefits of the BA model is that it is restricted to just two opinions and, correspondingly, three possible states: A, B, and the mixed opinion state AB. A simulation using these rules can be used to observe the effects of two competing opinions in a society, eventually converging to a consensus on one opinion. The simplicity of this model also allows for studying with it how spreading is affected by such phenomena as the addition of committed agents (nodes that will never change opinion regardless of interaction)^9^. Prior work on committed agent by J. Xie *et al*. put a minority population *p* of committed agents with a single opinion into a network with the remaining nodes holding the opposite opinion. Doing so introduces a phase transition in the system at *p*_*c*_ ≈ 0.1. Above *p*_*c*_, the system is in the absorbing regime and is pulled toward the consensus state which is reached in time on the order of *T*_*c*_ ∼ ln(*N*) where *N* is the network size. Below the critical point, however, the system will stay in an active steady state until some random extremely unlikely fluctuation pushes the system past a saddle point and into the absorbing regime, making the time to consensus to be on the order of *T*_*c*_ ∼ exp(*N*).

An extension of this work allows for the existence of two competing committed groups^10^, creating three distinct regions in parameter space designated as generalizing a region of the absorbing regime into a beak with respect to the committed populations for each opinion. The first region, with a high ratio of *p*_*A*_ with respect to *p*_*B*_, lies below the beak and represents regime absorbing towards the steady state for majority *A*. The second has a high ratio of *p*_*B*_ with respect to *p*_*A*_, lying above the beak and staying at the steady state for *B* majority. The beak region with a more even ratio of *p*_*A*_ to *p*_*B*_, is associated with a saddle point of the system that allows it to switch between the two steady states.

Further extensions of the committed BA models have become rather popular^11^,^12^,^13^,^14^, but most of them retain the infinite commitment of individuals. Here, we introduce and systematically examine impact of extensions of notion of commitment, from making it finite, so it can be lost, but also gained and finally its strength varied among the population according to some distribution. We start with an extended analysis of Galehouse *et al*. *waning commitment* model which accounts for human tendency to lose the commitment over time. In this model, it happens after a committed node hears *w* consecutive opposing opinions. This property causes an increase in *p*_*c*_ which becomes a function of *w: p*_*c*_(*w*) − *p*_*c*_(∞)∼*w*^*d*^, where *p*_*c*_(∞) represents a critical value with infinite commitment strength, *d* ≈ −1.73. When waining commitment is applied to competing committed groups, the beak widens in parameter space.

Here we seek to extend the various committed agent models^9^,^10^,^15^,^16^ by investigating further the effects of variable commitment as well as of heterogeneity of the commitment nodes on spread and stability of opinion in a network.

## Waning Commitment

The model of *waning commitment* first proposed in ref. 15 can be further examined via mean field analysis to investigate the impact of commitment loss and to analyze the model for large values of *w*. This model follows the rules of the binary agreement model with the addition of committed agents that are each assigned a commitment strength *w*; a node receiving *w* consecutive opposite opinions becomes uncommitted. In the original binary agreement model there are only two opinions (A and B) and only three states (*A*,*B*, and *AB*, where AB represents a neutral state). The addition of commitment strength, however, creates multiple substates for committed nodes corresponding to how close they are to losing their commitment, ie *A*_0_, 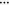, *A*_*w*_, *B*_0_, …, B_*w*′_ where the commitment strength for nodes in opinion A is *w*, and the commitment strength for nodes in opinion B is 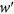. For simplicity, unless otherwise stated it is assumed that *w* = *w*′. Supplementary Fig. S1 shows the transitions between the substates.

## Increasing and Variable Commitment

The other side of *waning commitment* is the scenario where individuals have the ability to gain commitment through consecutive interactions affirming their current opinion. Here, the system is no longer initialized with some set population of committed minorities, but instead with some individuals having the capacity to become committed during the simulation. For simplicity (and to make it analogous to prior work), each individual with the capacity to commit will only be able to do so for a single opinion, set beforehand. This means there will be three general types of nodes: those that have no capacity to commit, those that have the capacity to commit to *A*, and those that have the capacity to commit to *B*. The agents currently holding opinion A and able to commit to A (*A*_0<*a*>_) will become committed (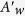) after consecutively receiving the opinion A *w* times; likewise for agents holding opinion *B*. The substate transitions are shown in the Supplementary Fig. S3. Finally, here we also introduce a new symmetric model with *variable commitment*, where nodes can enter or exit states of commitment based on the opinions they hear. The substate transitions are shown in the Supplementary Fig. S4.

## Distributed Commitment

In *variable commitment* model, commitment is not innate property of a node, instead it can be acquired or lost as a result of consecutive interactions with the same opinion. This allows for a more fluid commitment model. It can be made even more sophisticated by creating varying degrees of commitment strength for individual nodes to account for the tendencies for some people to be more stubborn or flexible with their ideas. This is novel because all prior analysis assumed that every committed node has the same level of commitment. *Distributed commitment* accounts for the diversity of people by allowing each committed node to have a personal commitment value assigned according to a distribution assumed for the given system.

## Agent Selection

The final extension of a commitment model presented here investigates selection of committed agents based on their network connections rather than randomly. The effects of such selection on the critical system parameters can advance our understanding of how committed agents may work in real systems, as well as our ability to design efficient strategies for creating consensus in the presence of committed agents.

## Results

### Waning Commitment

#### Mean Field Analysis

**Figure 1:**
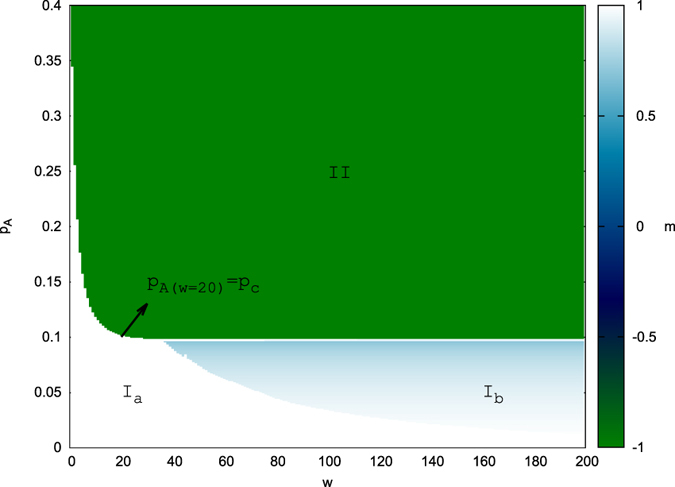
*Mean-field* diagram of the *waning commitment naming game* with single committed group. The quantity *m* is calculated when the steady state condition of a system is satisfied. The system starts with the initial values of *a*_0_ = *c* = 0, *b*_0_ = 1 − *p*_*A*_. Three regions of fixed points, *I*_*a*_, *I*_*b*_ and *II* are shown and discusses in the text.

**Figure 2:**
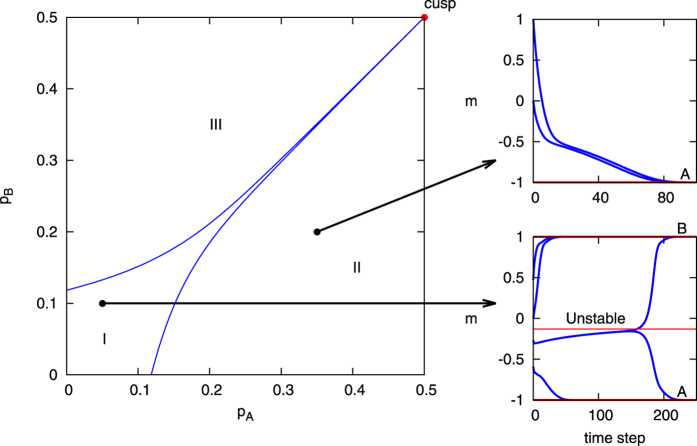
*Mean-field* diagram of the *waning commitment naming game* with two competing committed groups. The plots are calculated by the *mean-field* equations with commitment strength *w* = 10. (1) The left plot is a phase diagram containing three regions, in which the system has different fixed points. (2) The right plots show trajectories of points in regions I and II. The upper right plot is for the case where *p*_*A*_ = 0.35 and *p*_*B*_ = 0.2. The lower right plot is for the case where *p*_*A*_ = 0.05 and *p*_*B*_ = 0.1.

By deriving the probabilities of each state transition the system can undergo, *mean-field* equations are created leading to the critical values for the fraction of committed agents necessary to cause a phase transition in the system. As in the *constant commitment naming game*, each critical value represents a boundary separating two regions with different fixed points. Each can be found by using the bisection method on the *mean-field* equations as shown in Supplementary Table S2, yielding *p*_*A*_ in the single committed group model (shown in Fig. 1) and (*p*_*A*_, *p*_*B*_) in the two competing committed group model (shown in Fig. 2). The precision of this computation is 10^−10^ due to using the standard 32 bit arithmetic and limited time for computation.

With a single committed group if their fraction is higher than the critical fraction, there is only one fixed point corresponding to an absorbing state for the committed opinion. In the opposite case, when fraction of committed agents is below the critical fraction, there are three fixed points, a steady state for the uncommitted opinion, a saddle state, and an absorbing state for the committed opinion. Importantly, though, *at* the critical point *p*_*c*_, the steady state and the saddle state merge if the system is initialized with all non-committed nodes initially carrying the non-committed opinion. Figure 1 shows these two distinct regions separated by critical values of *p*_*A*_. The quantity 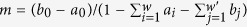 is an order parameter quantifying the degree of dominance of one opinion over the other once the system has reached a steady state.

As shown in Fig. 1, in the first region (regions I_*q*_ and I_*b*_), the system has three fixed points: two steady states and one unstable state. The active steady state is defined by inequalities 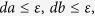 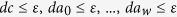 with *ε* = 10^−5^. Under this definition, the active steady state appears in region I_*b*_, which only occurs for the systems with high *w*. For lower values of *w* or *p*_*A*_, the system is in the I_*a*_ region where it reaches a consensus for state *B*. Lastly, when the value of *p*_*A*_ is high enough the system enters region II, where there is only a single fixed point for the consensus state *A*.

In the model with two competing committed minorities, three regions arise: one with no dominance for either opinion, one with dominance for opinion A, and one with dominance for opinion B. Figure 2 shows these three different regions separated by a “beak” of critical values in parameter space. The first region (region I) is within the beak, where the system has three fixed points corresponding to a consensus state for each of the two opinions as well as an unstable saddle state between them. Here the system can reach either consensus state depending largely on the initial conditions for the non-committed groups in the system (*a*_0_, *b*_0_ and *c*), or in the case of a relatively balanced beginning state can stay in the third unstable state until a random fluctuation pushes it into a consensus state. In the second and third regions (regions II and III), there is only a single fixed point for a consensus on the dominant committed opinion. Here the system will reach the consensus for the dominant opinion regardless of the initial conditions of the non-committed groups. The last area of interest in the case of competing committed agents is on the boundaries of the three regions, on which one of the consensus states and the unstable state merge together. This can only occur when all of the non-committed agents initially hold an opinion contrary to the more dominant committed side; i.e., at the points on the boundary between regions I and II, the initial values of *a*_0_ = 0, *c* = 0, *b*_0_ = 1−*p*_*A*_−*p*_*B*_ are needed; while for regions I and III, the needed initial values are *b*_0_ = 0, *c* = 0, *a*_0_ = 1−*p*_*A*_−*p*_*B*_. These boundaries meet at the cusp of the beak, where *p*_*A*_ = *p*_*B*_ = 0.5. At the cusp, all three states merge together.

#### Single Committed Group Critical Value Function

When there is only a single committed group in the system, the critical value of committed agents depends heavily on the commitment strength, *w*. The approximate value of *p*_*c*_ for specific values of *w* can be described as a critical value function *p*_*c*_(*w*) obtained via a simplified version of the mean field equations and approximated values for the infinite *w* system denoted *p*_*c*_(∞) and the time *t*_*w*_ needed to reach the merged state:


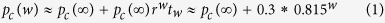


where *r* is a constant derived from Eq. 4 and simulation results with large *w*, as explained in the discussion of Eq. 4.

**Figure 3:**
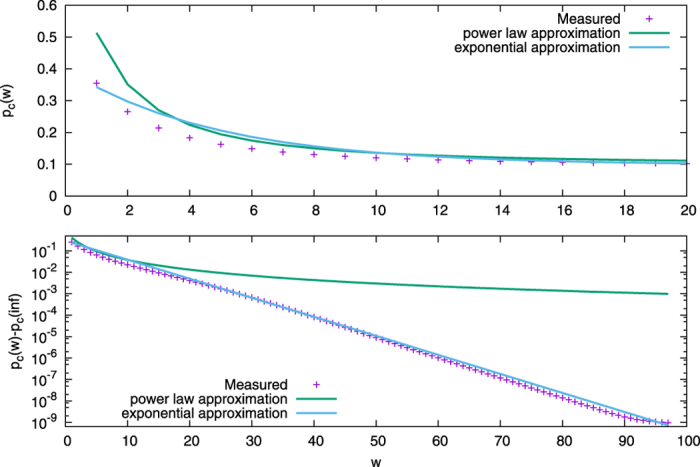
The critical value function of the *waning commitment naming game*. The top plot shows critical values of the fraction of committed agents with different commitment strengths. The bottom plot shows the differences between *p*_*c*_(*w*) and *p*_*c*_(∞). The critical value function is an exponential one especially when the commitment strength is large.

To ascertain validity of the approximate *p*_*c*_(*w*) given by Eq. 1, in Fig. 3, we compare it to the value measured by the bisection method with equations in Supplementary Table S2 as well as previously proposed function by Galehouse *et al*. in ref. 15. The latter is a power law, and thus *p*_*c*_(*w*) = *p*_*c*_(∞) + *a*(*b* + *w*^*d*^), where 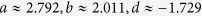. This function fits well for low values of *w*, but deviates strongly for high values. In contrast, the exponential function *p*_*c*_(*w*) = *p*_*c*_(∞) + *k*_*q*_^*w*^ fits very well for all values of *w*, when *k* ≈ 0.3, *q* ≈ 0.815.

### Increasing Commitment

#### Mean Field Analysis

**Figure 4:**
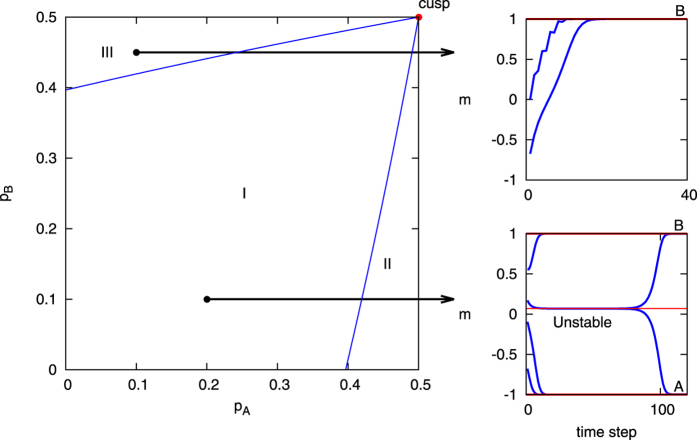
*Mean-field* diagram of the *increasing commitment naming game* with two competing committed groups. The plots are calculated by the *mean-field* equations with commitment strength w = 10. (1) The left plot is a phase diagram containing three regions. (2) The right plots show trajectories of points in region I and III. The top right is the case where (*p*_*A*_ = 0.1, *p*_*B*_ = 0.45) and the bottom right is the case where (*p*_*A*_ = 0.2, *p*_*B*_ = 0.1).

Many of the qualitative aspects of the critical values in the *increasing commitment* model mirror those in the *waning commitment* case. As before, when there is only a single committed group the system undergoes a phase transition at some critical value *p*_*c*_(*w*), defined by a critical value equation similar to Eq. 1. Also as before, in model of the two competing committed minorities, three regions arise separated by a beak. The fixed points of the system still change depending on the region as can be seen in the phase diagram in Fig. 4. Here, the value of *m* is adjusted slightly to fit the new model: 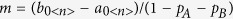.

The first region is inside the beak and has three fixed points: a steady state for the dominant opinion, a saddle point, and a steady state for the unstable opinion. Now, however, the addition of *increasing commitment* allows these steady state points to be either consensus (absorbing) or active steady states, depending on whether any of the nodes achieved full commitment. The trajectories still show the system able to reach either both absorbing states or one stable and one unstable state. In the second and third regions, the system has only the single steady state fixed point for the dominant opinion. As before, the systems trajectory will force it towards this state regardless of other initial parameters. Finally, the last point of interest is the shape of the boundaries, which have lost all curvature and are linear in parameter space.

#### Single Commited Group Critical Value Function

**Figure 5:**
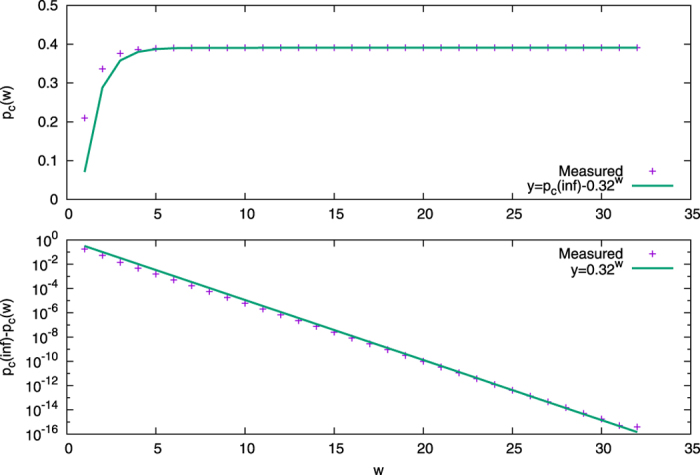
The critical value function of the *increasing commitment naming game*. The top plot shows critical values of the fraction of committed agents with different commitment strengths. The bottom plot shows the differences between *p*_*c*_(*w*) and *p*_*c*_(∞). Both plots shows our function fits data well.

The analysis of a critical value function for the case with a single committed group is also similar to that done for the *waning commitment* model. By applying bisection to the mean field equations, the real critical values can be obtained. Specifically, the general form is 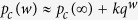 where 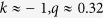. Figure 5 shows the critical value function with this added commitment strength parameter. With the limitation of computing precision around 10^−16^, the computed critical value of 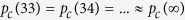. Since we cannot get critical values for *w* > 33 with higher precision, in the experiment, we consider *w* = 33 as computed infinite commitment strength.

### Distributed Commitment

#### Critical Value for a Single Committed Group

As seen above, using a uniformly distributed *variable commitment* alters the value of *p*_*c*_ as 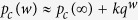 where *k* > 0 and *q* ≈ 0.815 for the *waning commitment* model, while *k* < 0 and *q* ≈ 0.32 in the *increasing commitment* model. The most straightforward way to incorporate distributed commitment into these models is to simply use the average commitment strengths in these formulas. Indeed, there is a good deal of legitimacy to this method, as it can be verified by estimating the critical value to be 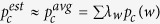 where *λ*_*w*_ is the fraction of committed agents with commitment strength *w*, then comparing to the real critical values for systems with uniformly distributed commitment strengths. The main quantities of interest in this comparison are the relative errors for both the *waning commitment* model and the *increasing commitment* model. These quantities can be seen in Supplementary Fig. S5, where the relative errors are around 1% for small 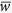, and negligible for large 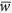. It also should be noted that since a group of distributed values can be represented by multiple subgroups of uniformly distributed values, then if the estimation is accurate for the uniform distribution it is very likely to be accurate for other distributions.

#### Effects of Standard Deviation on the Critical Value Function

Using the mean value estimation above, the effect of increasing standard deviation on the critical value function can be ascertained by studying the effect of a unitary increase in standard deviation on a system with an arbitrary distribution of commitment strengths. Doing so yields Eq. 9, which makes it clear that *k* is the controlling parameter of whether *p*_*c*_ is growing or shrinking with standard deviation. When *k* > 0, 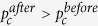 and thus the critical population is growing with increased standard deviation. When *k* < 0, however, 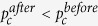 and thus the critical population shrinks with increased standard deviation. As mentioned before, the *waning commitment* model has *k* > 0 while the *increasing commitment* model has *k* < 0. Therefore the two models react in opposite ways to an increase in standard deviation, with *waning commitment* growing its critical population while *increasing commitment* shrinking it.

#### Competing Committed Minorities

To test the validity of our analysis on systems with distributed commitment, we compare them to Monte Carlo simulations. Those can be done for any type of commitment distribution, so for completeness we compare four different types of systems: constant value, uniform distribution, normal distribution, and power-law distribution. For simplicity, for a single simulation, all committed agents follow the same distribution regardless of their opinion.

For the case where commitment strengths, *w* is set to 10, which also serves as the mean for all the other distributions. The uniform distribution has a width of 18 (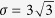). The normal distribution uses *w* ∈ [1, 19] and variance *σ*^2^. Lastly, the power law follows 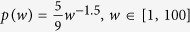, which has σ = 16.3.

**Figure 6:**
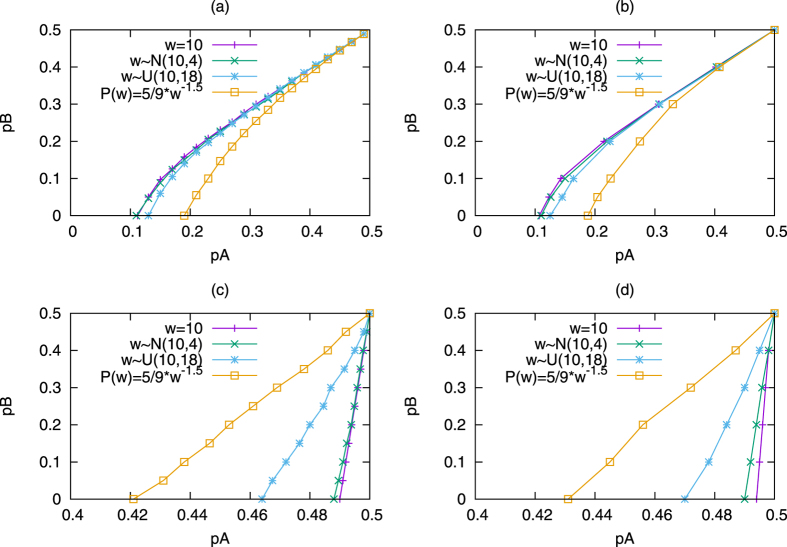
The critical value curves comparison. All the experiments are based on networks generated by Barabasi-Albert (BA)^17^ and Erdos-Renyi (ER) models with parameters *N* = 1000, *m*_0_ = *m* = 5, *p* ≈ 0.01, where the average degree of each network is 10, the mean of commitment strengths 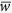 of each distribution is 10. The standard deviation in each case is 0, 2, 5.196 and 16.3 for constant value, and then distributed normally, uniformly and according to power-law commitment strengths. Each critical value is averaged over 100 runs, half runs reaches consensus state A, while the other half reaches consensus state B. For simplicity, we just plot the critical values when *p*_*A*_ > *p*_*B*_. Subfigures (**a**) and (**b**) show the critical value curves in the *waning commitment naming game. pA, pB* are the initial fractions of committed agents at state *A, B*. Subfigures (**c**) and (**d**) show the critical value curves in the *variable commitment naming game*. The *variable commitment* yields very similar results to *increasing commitment*, but runs much faster. *pA, pB* are the initial fractions of agents able to commit at state *A, B*, which are initialized as uncommitted, but are likely to become committed during interactions. Subfigures (**a**) and (**c**) are simulations in BA networks, while subfigures (**b**) and (**d**) are simulations in ER networks.

Based on simulations runs for each desired distribution of commitment strengths (constant commitment, uniform distribution, normal distribution, and power law distribution; all with 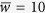) and then averaging the results yield the critical value curves for the committed populations shown in Fig. 6. For comparisons sake, these simulations are run on waning commitment (Fig. 6(a)) and variable commitment (Fig. 6(c)). Variable commitment is used instead of increasing commitment because the latter allows for infinitely committed nodes and often requires extremely large run times. Instead, using a variable commitment model where all the nodes with the ability to commit begin uncommitted (similar to the initial conditions of a pure increasing commitment simulation) allows for a close approximation because it forces the *increasing commitment* effect to occur before *waning commitment* is possible. This allows for the eventual loss of commitment to lower simulation time while maintaining much of the behavior of the *increasing commitment model*. In each case, as the standard deviation increases the critical value curve moves to the right in the *waning commitment naming game*, and to the left in the *variable commitment naming game*. In other words, the wider distributed is the commitment strengths of a group of committed agents (holding the same opinion), the easier it is for them to change their opinions. The exact opposite is true for a group of agents able to commit to an opinion. Since the standard deviation are different for different distributions in Fig. 6, we run experiments with different standard deviations and fixed distribution type in Supplementary Fig. S7, which yield exactly the same conclusions.

### Agent Selection

The previous sections show that the critical value is influenced by the variance of the commitment strength distribution. Thus, given a certain commitment strength distribution, there should be methods to enhance the ability of the committed agents to spread their opinion by placing high values of commitment on nodes with specific network properties.

The most direct way to go about this is to select the value of commitment strength based on the degree of an agent. This can be done in many different ways, but three of the most straight forward are matching by equal ranks, inverse ranks or random ranks of commitment strength and node degrees. Ranks are assigned by sorting agents in decreasing order of their degrees and commitments in decreasing order of their strength. All our previous experiments are based on the random matching.

**Figure 7:**
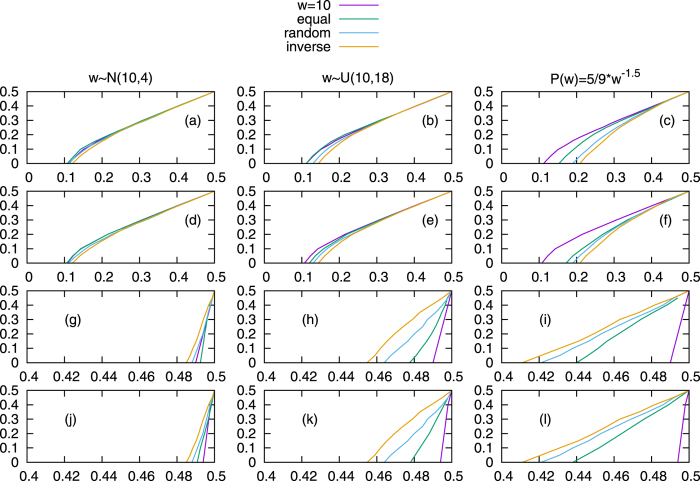
The critical value curves comparisons of matching equal, inverse or random ranks of node degrees and commitments. The committed agents are selected by three different strategies mentioned above. All the experiments are based on networks generated by Barabasi-Albert (BA) and Erdos-Renyi (ER) models with parameters *N* = 1000, *m*_0_ = *m* = 5, *p* ≈ 0.01, where the average degree of each network is 10, the mean of commitment strengths 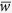 of each distribution is 10. Each critical value is averaged over 100 runs. For all subfigures, the x-axis is *p*_*A*_, the y-axis is *p*_*B*_. For simplicity, we just plot the critical values when *p*_*A*_ > *p*_*B*_. Subfigures (**a–f**) show experiments of the *waning commitment naming game*, while subfigures (**g–l**) show experiments of the *variable commitment naming game*. Subfigures (**a–c**) and (**g–i**) are simulations in BA networks, while subfigures (**d–f**) and (**j–l**) are simulations in ER networks.

Figure 7 shows the critical value curve comparisons among three matching methods. For each distribution in the *waning commitment naming game*, the agents committed to opinion A benefit from the equal rank matching (the critical values of *p*_*A*_ of equal rank is smaller than that of random rank), but suffer from the inverse rank matching. However, in the *variable commitment naming game*, the agents that are able to commit to A benefit from the inverse rank matching, but suffer from the equal rank matching.

## Discussion

### Waning Commitment

Analysis of the mean field equations for the *waning commitment* model culminates in the discovery of three fixed points: the A consensus state, the B consensus state, and an unstable state. This is analogous to the results previously discussed for the *constant commitment* model, where inside the “beak” in parameter space, there are three fixed points: a consensus state in which all uncommitted agents have the same opinion, a saddle point, and an active steady state without agreement^9^,^10^. The obvious differences are that in the *waning commitment* model, the consensus states become more complete (as the loss of commitment allows even previously committed nodes to change their opinion) and the active steady state becomes a consensus state for the same reasons. The similarities make sense, since the *constant commitment* model is in fact just a special case of the *waning commitment* model in which the commitment strength is infinite. In that sense, the *waning commitment* is a more general model for human behavior.

In the simpler case with only a single committed group, the analysis of the mean field equations leads to a new form for the dependence of *p*_*c*_ on *w*. This new function outperforms one proposed in previous work^15^ which is an average over multiple direct simulations for limited values of commitment strength and subject to the large experimental error inherent in direct simulation. Indeed, tests on the power law function proposed there for large values of *w* show great deviation from the values obtained via the bisection method. In contrast, the new exponential function proposed here is able to capture the behavior at large *w* while still remaining valid in the small *w* regime.

A final point of interest is the degree of validity of the approximations made in the bisection method. Due to computational precision of the mean field equations, *w* = 98 is approximated to be equivalent to infinite commitment strength. This approximation holds surprisingly well considering that there are around 200 equations to simulate. In fact, the error between *p*_*c*_(98) and *p*_*c*_(∞) is only *ε* = 9.0 * 10^−10^, when the precision is set to 10^−10^. This precision shows quite clearly that the analytic results obtained hold up to more exact values despite the difficulties of solving the intermediate equations corresponding to each of the substates of the system.

### Increasing Commitment

*Increasing Commitment* is largely proposed to mirror *waning commitment* and round out the subject to set up *variable commitment*. As expected, in many ways the increasing commitment model is directly opposite of the waning commitment model. In fact, in the single committed group model, the critical value function is formulated in the same way with only a different set of parameters: 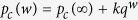. In the *waning commitment*, 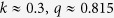 while in the *increasing commitment*, 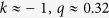. *k* represents the direction of the change of the committed opinion, while *q*^*w*^ represents the displacement. With the increase of *w*, the displacement decreases and finally shrinks to 0. The two results actually display a very nice symmetry, and the specifics of the parameters speak volumes about the general behavior of the system. Clearly, due to the way the models have been constructed, they help/hinder the critical population via the same mechanism leading to equations of the same form. Yet the differences in the parameters also show the degree to which these changes manifest themselves. Namely, since the *waning commitment* model has a *q* much closer to 1, it will likely be less sensitive to changes in *w*, while the fact that it is much higher than the *q* in the *increasing commitment* model implies that it will be more likely to deviate from *p*_*c*_(∞). This makes intuitive sense given the initial conditions of the system having a much higher density of nodes in opinion B than opinion A. When allowed to propagate, it is much more likely that a committed node will hear *w* consecutive opposing opinions than *w* consecutive confirming opinions. Thus, the special mechanism inherent to *waning commitment* is much more likely to occur than that in *increasing commitment*. Different initial conditions, however, can have very strong effects on the dynamics of the system. For instance, in the *variable commitment* naming game, it is shown that the *increasing commitment* effect can be allowed to dominate simply by initializing all of the nodes with the ability to commit into an uncommited state so that they are forced to undergo the *increasing commitment* effect before there is even a possibility for *waning commitment* to take place. It is clear that alterations on the initial conditions can change the system dramatically, and defining them clearly is extremely important. In this case, initial conditions were chosen in a natural way to fit the goals of each experiment.

### Distributed Commitment

The idea behind distributed commitment is to create a system with a more human-like heterogeneity in people’s willingness to change. In doing so, it becomes apparent that for the most part systems of differently distributed commitment values will behave similarly to our systems above, only with the constant *w* replaced by the average value of *w* across the system. Systems with the same mean do not behave identically, though, as the critical values also have some dependence on the standard deviation of *w* across the system. Like before, the *increasing commitment* and *waning commitment* cases behave in opposite ways: when the standard deviation increases, the critical values in the *waning commitment* model also increase while in the *increasing commitment* model the critical values decrease. These results can be confirmed by using Jensen’s Inequality^18^ (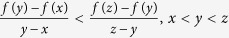) given the strictly convex critical value function of *waning commitment* and the strictly concave critical value function of *increasing commitment* (for details see Supplementary materials). The observed behavior can be explained by considering high and low standard deviation on a node by node basis: high standard deviation will lead to some deeply committed nodes and some easily swayed nodes. For the *waning commitment model*, this means that many of the nodes are practically not committed at all; they will quickly lose their commitment and become normal nodes, leaving only the deeply committed nodes to try and dominate the system. This will obviously increase the value of *p*_*c*_, as the committed population will, in effect, be only a fraction of those initially designated. On the other hand in the *increasing commitment* model, having these easily swayed nodes is a very good thing. Considering the relative rarity of the switching events in this model discussed earlier, having some group easily switched can create a strong foothold for the committed opinion early on in the simulations, decreasing the initial number of committed agents necessary to create consensus.

### Agent Selection

The final tests of variably committed individuals establishes how the locations of the committed agents within the structure of the network impact the critical values of the systems. By designating nodes of higher/lower degree as committed agents, large changes in the critical values can be induced. This is an idea often tested on prior models of committed nodes^5^, leading to the conclusion that “selecting a small number of the nodes with the highest degree works best” for a committed minority to maximize their influence. Indeed, this can still be seen in the case of variable commitment. However, since the seed selection problem in the Naming Game has not been fully analyzed, a more in depth study is left for future work.

## Methods

### Waning Commitment

#### Mean Field Analysis

Supplementary Table S1 shows the change in the number of agents at each substate after one system interaction. In the *mean-field* analysis, each interaction is considered a system time step, differing from simulations on *BA* and *ER* networks where *N* interactions are considered a system time step (where *N* is the network size).

To simplify the equations, we define the quantity *r* as *r* ≡ *b* + *c*/2, following the approach used in ref. 19, where *a, b, c* represent the fraction of agents at states *A, B, AB*. In order to analyze the steady states of this naming game, we set all derivatives to zero, which yields the simple formula (for 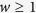)


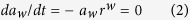


where *a*_0_, …, *a*_*w*_, *b*_0_, …, *b*_*w*′_ represent the fraction of agents at substates A_0_, …., *A*_*w*_, *B*_0_, …, *B*_*w*′_. This equation implies that for finite values of *w*, either *a*_*w*_ = 0 or *r* = 0, but applying these equations recursively reveals 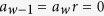, and thus *a*_1_ = … = *a*_*w*−1_ = 0 (and similarly *b*_1_ = … = *b*_*w′*−1_ = 0). This leads to a final set of equations to be solved:


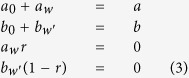


Solving these equations under the assumption that 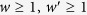 gives three possible fixed points:

- If *r* = 0; then *a* = 1, *b* = *c* = 0, yielding a consensus state on A.
- If *r* = 1; then *b* = 1, *a* = *c* = 0, yielding a consensus state on B.
- If 0 < *r* < 1; then 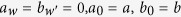, due to the rate equations 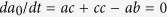 and *db*_0_/*dt* = *bc* + *cc* − *ba* = 0 under the restriction *a* + *b* + *c* = 1. Solving simply yields 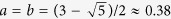, 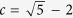, an unstable fixed point.

#### Single Committed Group Critical Value Function

By using the mean field equations in Supplementary Table S1 in conjunction with the previously stated fact that committed agents are lost at a rate of 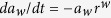, it can be deduced that after *t* interactions the total number of committed agents will be approximately 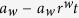. When p_*A*_ = *p*_*c*_, the saddle state and steady state merge. Hence, we can assume that after times 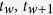 for large values of *w*, systems with commitment strengths *w* and *w* + 1 will be close to the merged state (with the similar number of remaining committed agents). Therefore


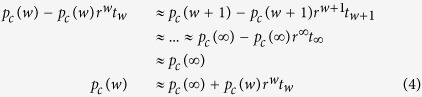


With computing precision at 10^−10^, the computed critical value for 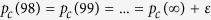, where *p*_*c*_(∞) = 0.09788910377 is the theoretical critical value of infinite commitment strength, *ε* = 9.0 * 10^−10^. Thus, with this precision *p*_*c*_(98) ≈ *p*_*c*_(∞), so *w* = 98 will be considered as the value of computed “infinite” commitment strength. The critical values are, as before, calculated using bisection on the mean field equations. When there is only a single committed minority and in the system initial state only the committed agents hold opinion A, the critical value is defined by Eq. 4: 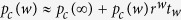. This can be made more exact by looking at the known values for the “infinite” commitment case. Comparing Eq. 4 with simulation results for p_*A*_ = *p*_*c*_(98), we find that the merged state occurs for *r* ≈ 0.728.

**Figure 8:**
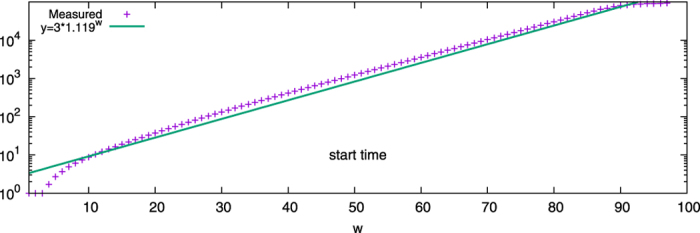
The start times of system with each commitment strength *w* at its critical point.

The value of *t*_*w*_ is much harder to ascertain, but can be approximated as the system start time, which represents the time from the initial state to the merged state. Three running time samples for *w* = 10, 30, 90 can be found as Supplementary Fig. S2, in which we distinguished three periods of which the first call start time, and denoted *t*_*w*_ is used in Eq. 4. Figure 8 shows the start times for systems with different *w*′s. For this analysis, it will be approximated as 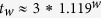, thus 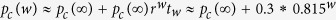.

### Increasing Commitment

Here *A*_0_, *AB*, and *B*_0_ represent that agents currently holding opinion A, AB, B; *A*_0<*a*>_ represents agents currently holding opinion A and able to commit to A (*A*_0<*b*>_ for those in A able to commit to B); and *A*_0<*n*>_, *AB*_<*n*>_, *B*_0<*n*>_ represent normal agents currently holding opinion A, AB, B respectively. The values 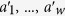, 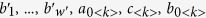 represent the fraction of agents at substates 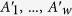, 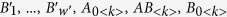.

#### Mean Field Analysis

Supplementary Table S3 shows, the change of numbers of agents able to commit to A at each substate after one system interaction. In order to analyze the steady states of this naming game, we set all derivatives to zero, and solve the equation set. If 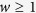,


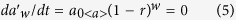


Applying this recursively for finite 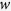 leads to 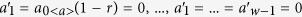. Similarly, 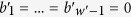. 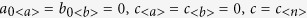. This leads to a final set of equations to be solved:


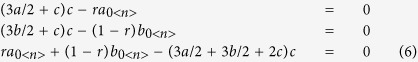


Once solved, these equations give three possible solutions for the system

- If *r* = 0; then *a* = 1, *b* = *c* = 0, and 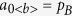. The system reaches a consensus state on A.
- If *r* = 1; then *b* = 1, *a* = *c* = 0, and 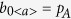. The system reaches a consensus state on B.
- If 0 < *r* < 1; 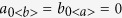, 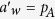, and 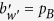. The system may reach a consensus state, an active steady state or an unstable state.

where 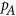, 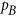 represent the total fraction of agents with potential to commit to A, B.

### Distributed Commitment

#### Effects of Standard Deviation on the Critical Value Function

The impact of changes of standard deviation on the critical values of variable commitment model can be ascertained quite generally using an arbitrary distribution of commitment strengths. Using the mean value estimation described in the Results section, the estimated critical value of the system can be written as


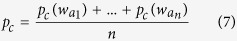


where there are 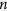 committed agents and the commitment strength of each agent is 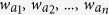. Any increase in standard deviation preserving average of integer commitments can be decompose into a series of pairs of changes 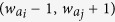 where 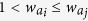 each pair making the same change. Indeed, after a pair of changes, 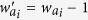 and 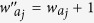. To find the effect of this change, the value of 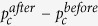 must be calculated using Eq. 7. Doing so yields


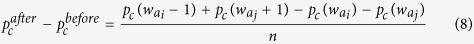


which after plugging in the exponential form of 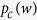 becomes


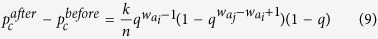


## Additional Information

**How to cite this article**: Niu, X. *et al*. The impact of variable commitment in the Naming Game on consensus formation. *Sci. Rep.* **7**, 41750; doi: 10.1038/srep41750 (2017).

**Publisher's note:** Springer Nature remains neutral with regard to jurisdictional claims in published maps and institutional affiliations.

## Supplementary Material

Supplemental Materials
