# Supplementary material for: The impact of variable commitment in the Naming Game on consensus formation
Source: Sci Rep. 2017 Feb 2;7:41750. doi: 10.1038/srep41750 (PMC5288711; doi:10.1038/srep41750)
Supplement: Supplemental Materials [file srep41750-s1.pdf]

Supplemental Materials for: the impact of variable commitment in the Naming Game on consensus formation

Xiang Niu,[1,2,*] Casey Doyle,[1,3] Gyorgy Korniss[1,3] Boleslaw K. Szymanski[1,2]

[1]Rensselaer Polytechnic Institute, Social Cognitive Networks Academic Research Center, Troy, NY, 12180, USA
[2]Rensselaer Polytechnic Institute, Department of Computer Science, Troy, NY, 12180, USA
[3]Rensselaer Polytechnic Institute, Department of Physics, Applied Physics, and Astronomy, Troy, NY, 12180, USA

[*]Correspondence and requests for materials should be addressed to X.N. (email: nx.niuxiang@gmail.com)

1 Waning Commitment

Figure S1 shows the transitions between the substates. Table S1 shows the change in the number of agents at each substate after one system interaction, where a, b, c represent the fraction of agents at states A, B, AB and $a_0, ..., a_w, b_0, ..., b_{w'}$ represent the fraction of agents at substates $A_0, ..., A_w, B_0, ..., B_{w'}$. The notation $A(50\%)$ denotes the case in which the speaker has 50% chance to send the opinion A during the interaction. After combining all of the *mean-field* equations in Table S1, we arrive at the equations in Table S2.

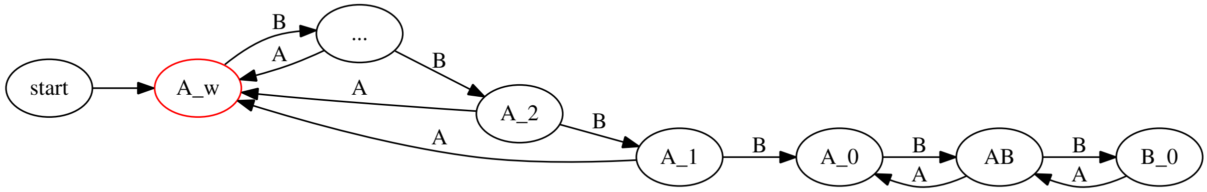

Figure S1: Substates transition graph of the *waning commitment naming game*. We use A, B, AB to represent the current state of an agent. The commitment strength of agents holding opinion A is w, B is w'. $A_0, A_1, ..., A_w$ are substates of agents at state A, where A_0 represent an uncommitted agent holding opinion A, A_w represent a fully committed agent holding opinion A, A_i represent a committed A agent, who has already consecutively received the opposite opinion $w - i$ times, and will become uncommitted after it continues consecutively receiving the opposite opinion another i times.

1.1 Saddle Point

There are three fixed points in the *waning commitment* model:

- If $r = 0$; then $a = 1, b = c = 0$, yielding a consensus state on A.
- If $r = 1$; then $b = 1, a = c = 0$, yielding a consensus state on B.
- If $0 < r < 1$; then $a_w = b_{w'} = 0, a_0 = a, b_0 = b$ due to the rate equations $da_0/dt = ac + cc - ab = 0$ and $db_0/dt = bc + cc - ba = 0$ under the restriction $a + b + c = 1$.
  Solving these equations yields $a = b = (3 - \sqrt{5})/2 \approx 0.38$, $c = \sqrt{5} - 2$, an unstable fixed point.

Before Interaction	Opinion	After Interaction	*Mean-Field*
$A->A_0$	A	$A-A_0$	
$A->A_i, i \in [1, w-1]$	A	$A-A_w$	$\frac{da_i}{dt}=-a*a_i, \frac{da_w}{dt}=a*a_i$
$A->A_w$	A	$A-A_w$	
$A->B_0$	A	$A-AB$	$\frac{db_0}{dt}=-a*b_0, \frac{dc}{dt}=a*b_0$
$A->B_1$	A	$A-B_0$	$\frac{db_1}{dt}=-a*b_1, \frac{db_0}{dt}=a*b_1$
$A->B_{j'}, j' \in [2, w']$	A	$A-B_{j'-1}$	$\frac{db_{j'}}{dt}=-a*b_{j'}, \frac{db_{j'-1}}{dt}=a*b_{j'}$
$A->AB$	A	$A-A_0$	$\frac{dc}{dt}=-a*c, \frac{da_0}{dt}=a*c$
$B->A_0$	B	$B-AB$	$\frac{da_0}{dt}=-b*a_0, \frac{dc}{dt}=b*a_0$
$B->A_1$	B	$B-A_0$	$\frac{da_1}{dt}=-b*a_1, \frac{da_0}{dt}=b*a_1$
$B->A_j, j \in [2, w]$	B	$B-A_{j-1}$	$\frac{da_j}{dt}=-b*a_j, \frac{da_{j-1}}{dt}=b*a_j$
$B->B_0$	B	$B-B_0$	
$B->B_{i'}, i' \in [1, w'-1]$	B	$B-B_{w'}$	$\frac{db_{i'}}{dt}=-b*b_{i'}, \frac{db_{w'}}{dt}=b*b_{i'}$
$B->B_{w'}$	B	$B-B_{w'}$	
$B->AB$	B	$B-B_0$	$\frac{dc}{dt}=-b*c, \frac{db_0}{dt}=b*c$
$AB->A_0$	$A(50\%)$	A_0-A_0	$\frac{dc}{dt}=-c*a_0/2, \frac{da_0}{dt}=c*a_0/2$
$AB->A_i, i \in [1, w-1]$	$A(50\%)$	A_0-A_w	$\frac{dc}{dt}=-c*a_i/2, \frac{da_0}{dt}=c*a_i/2, \frac{da_i}{dt}=-c*a_i/2, \frac{da_w}{dt}=c*a_i/2$
$AB->A_w$	$A(50\%)$	A_0-A_w	$\frac{dc}{dt}=-c*a_w/2, \frac{da_0}{dt}=c*a_w/2$
$AB->B_0$	$A(50\%)$	$AB-AB$	$\frac{db_0}{dt}=-c*b_0/2, \frac{dc}{dt}=c*b_0/2$
$AB->B_1$	$A(50\%)$	$AB-B_0$	$\frac{db_1}{dt}=-c*b_1/2, \frac{db_0}{dt}=c*b_1/2$
$AB->B_{j'}, j' \in [2, w']$	$A(50\%)$	$AB-B_{j'-1}$	$\frac{db_{j'}}{dt}=-c*b_{j'}/2, \frac{db_{j'-1}}{dt}=c*b_{j'}/2$
$AB->AB$	$A(50\%)$	A_0-A_0	$\frac{dc}{dt}=-c*c, \frac{da_0}{dt}=c*c$
$AB->A_0$	$B(50\%)$	$AB-AB$	$\frac{da_0}{dt}=-c*a_0/2, \frac{dc}{dt}=c*a_0/2$
$AB->A_1$	$B(50\%)$	$AB-A_0$	$\frac{da_1}{dt}=-c*a_1/2, \frac{da_0}{dt}=c*a_1/2$
$AB->A_j, j \in [2, w]$	$B(50\%)$	$AB-A_{j-1}$	$\frac{da_j}{dt}=-c*a_j/2, \frac{da_{j-1}}{dt}=c*a_j/2$
$AB->B_0$	$B(50\%)$	B_0-B_0	$\frac{dc}{dt}=-c*b_0/2, \frac{db_0}{dt}=c*b_0/2$
$AB->B_{i'}, i' \in [1, w'-1]$	$B(50\%)$	$B_0-B_{w'}$	$\frac{dc}{dt}=-c*b_{i'}/2, \frac{db_0}{dt}=c*b_{i'}/2, \frac{db_{i'}}{dt}=-c*b_{i'}/2, \frac{db_{w'}}{dt}=c*b_{i'}/2$
$AB->B_{w'}$	$B(50\%)$	$B_0-B_{w'}$	$\frac{dc}{dt}=-c*b_{w'}/2, \frac{db_0}{dt}=c*b_{w'}/2$
$AB->AB$	$B(50\%)$	B_0-B_0	$\frac{dc}{dt}=-c*c, \frac{db_0}{dt}=c*c$

Table S1: The list of all interactions of the *waning commitment naming game*. a, b, c represent the fraction of agents at states A, B, AB. $a_0, ..., a_w, b_0, ..., b_{w'}$ represent the fraction of agents at substates $A_0, ..., A_w, B_0, ..., B_{w'}$

Derivatives	*Mean-Field*	Let $r = b + c/2 = (a-1)(a-2)/2$
$da_0/dt = 0$	$ac + cc - a_0 b + (a - a_0)c/2 + a_1(b + c/2) = 0$	$ac + cc - a_0 b + (a - a_0)c/2 + a_1 r = 0$
$da_1/dt = 0$	$-a_1(a + b + c) + a_2(b + c/2) = 0$	$-a_1 + a_2 r = 0$
...	...	...
$da_{w-1}/dt = 0$	$-a_{w-1}(a + b + c) + a_w(b + c/2) = 0$	$-a_{w-1} + a_w r = 0$
$da_w/dt = 0$	$(a_1 + ... + a_{w-1})(a + c/2) - a_w(b + c/2) = 0$	$(a_1 + ... + a_{w-1})(1 - r) - a_w r = 0$
$db_0/dt = 0$	$bc + cc - b_0 a + (b - b_0)c/2 + b_1(a + c/2) = 0$	$bc + cc - b_0 a + (b - b_0)c/2 + b_1 r = 0$
$db_1/dt = 0$	$-b_1(a + b + c) + b_2(a + c/2) = 0$	$-b_1 + b_2(1 - r) = 0$
...	...	...
$db_{w'-1}/dt = 0$	$-b_{w'-1}(a + b + c) + b_{w'}(a + c/2) = 0$	$-b_{w'-1} + b_{w'}(1 - r) = 0$
$db_{w'}/dt = 0$	$(b_1 + ... + b_{w'-1})(b + c/2) - b_{w'}(a + c/2) = 0$	$(b_1 + ... + b_{w'-1})r - b_{w'}(1 - r) = 0$

Table S2: Overall derivatives of the *waning commitment naming game*.

The first two points are relatively straightforward, but the third requires some further investigation to determine its exact nature. To confirm the instability of the fixed point, the system can be examined at some point near the unstable saddle point. If

$$\begin{aligned} a_0 &= a = sp + \delta_1 \\ b_0 &= b = sp + \delta_2 \end{aligned} \tag{S1}$$

where $sp = (3-\sqrt{5})/2$, and using previously defined rates of change (ie $a+da/dt = a+ac+cc-ab = (1-b)^2$) it can be shown that

$$\begin{aligned} da/dt' &= (a + da/dt) + d(a + da/dt)/dt - a \\ &= (1 - (1 - a)^2)^2 - a \\ &= a(a - 1)\delta_1(a - sp') \\ db/dt' &= (b + db/dt) + d(b + db/dt)/dt - b \\ &= b(b - 1)\delta_2(b - sp') \end{aligned} \tag{S2}$$

where $sp' = (3 + \sqrt{5})/2, 0 \leq a \leq 1, 0 \leq b \leq 1, \frac{da/dt'}{\delta_1} \geq 0, \frac{db/dt'}{\delta_2} \geq 0$. Therefore, for $\delta_1 > 0$, a will strictly increase until it becomes one while for $\delta_1 < 0$ it will strictly decrease until it reaches zero.

Further, the case where $a = b \neq (3 - \sqrt{5}/2)$ (where the system parameters are equal yet not in the saddle state described above) bears investigation. In case when $\delta_1 = \delta_2 > 0$, both a and b will grow and become 0.5 due to their equality and constraint $0 \leq a + b \leq 1$. Then in the next step, $a + da/dt = (1 - b)^2 = 0.25$, $b + db/dt = (1 - a)^2 = 0.25$ from which a and b will grow back to 0.5. In the opposite case where $\delta_1 = \delta_2 < 0$, the same limits on a and b values will apply. This forces the system into a trajectory along which the system moves back and forth between $a = b = 0.25$, and $a = b = 0.5$. In practice, however, this trajectory is unstable similarly to the one described above. If at any step δ_1, δ_2 get unequal, the system will veer off this trajectory.

1.2 Running Time

Figure S2 shows three running periods (start time, unstable time and escape time) for three samples values of waning ($w = 10, 30, 90$). A is a point where initial p_A is slightly greater than $p_c(w)$, while B is a point where initial p_A is slightly smaller than $p_c(w)$. The end of start time is a time step when the value of r is closest to 0.728. For most of cases, system reaches an unstable state and then escape from it ending in consensus state of either A or B; for $w = 90$, system reaches either consensus state on A or an active steady state for B.

The results of the running time analysis show that around the merged point, the escape time of A is constant for different values of w, while the escape time of B grows exponentially with w. Intuitively this can be explained by the process of the system transitioning to consensus on A, during which the value of w does not matter. The transition time only measures the speed of B shifting to A. During the process of transitioning to consensus on B, w matters because the higher w, the more time is needed for committed nodes with opinion A to lose their commitment. It should be noted that when w is infinite, this transition time tends to infinity, indicating that in the infinite commitment limit the steady state for B can not be reached. Instead, the case of infinite commitment forces an active steady state for B.

2 Increasing Commitment

Similarly to Fig. S1 and Table S1 for the *wanging commitment* model, Fig. S3 illustrates the transitions between the substates of the *increasing commitment* model; and Table S3 shows, after one system interaction, the change of numbers of agents able to commit to A at each substate. Here A_0, AB, and B_0 represent that agents currently holding opinion A, AB, B; $A_{0<a>}$ represents agents currently holding opinion A and being able to commit to A (similarly $A_{0<b>}$ denotes number of those in A being able to commit to B); and $A_{0<n>}, AB_{<n>}, B_{0<n>}$ represent normal agents that currently hold opinion A, AB, B respectively. The values $a'_1, ..., a'_w, b'_1, ..., b'_{w'}, a_{0<k>}, c_{<k>}, b_{0<k>}$

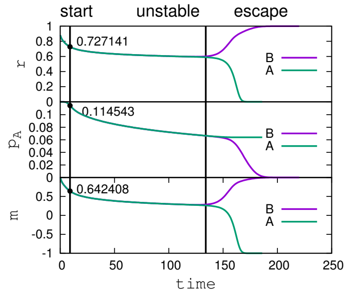

(a) $w = 10$

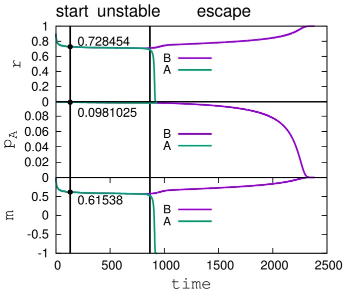

(b) $w = 30$

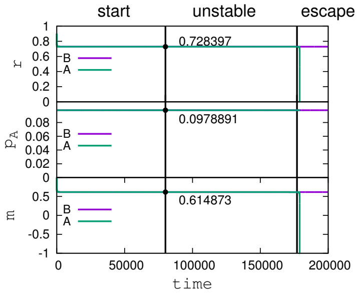

(c) $w = 90$

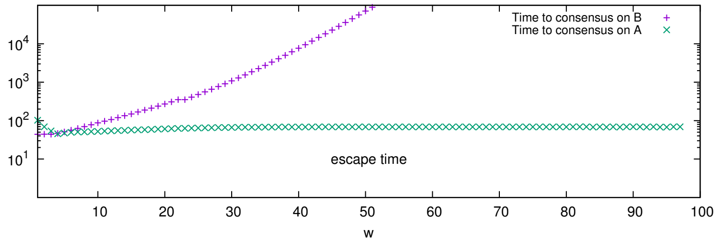

(d) escape time

Figure S2: Running time to merged state in which the steady state and the saddle state coincide. The time to transition from unstable state to consensus state on A is constant, while from unstable state to consensus state on B grows exponentially with w.

represent the fraction of agents at substates $A'_1, ..., A'_w$, $B'_1, ..., B'_{w'}, A_{0<k>}, AB_{<k>}, B_{0<k>}$. As before, after combining all of the equations in Table S3, we obtain equations for overall derivatives as shown in Table S4.

3 Distributed Commitment Strength

3.1 Estimated Critical Value

A straightforward approach is to use the average critical values of each commitment strength. Denoting the fraction of committed agents with commitment strength w by λ_w, so $\sum \lambda_w = 1$, the estimated critical value then becomes

$$p_c^{est} \approx p_c^{avg} = \sum \lambda_w p_c(w) \tag{S3}$$

To verify the accuracy of the estimation, we calculate the real critical values of systems with uniformly distributed commitment strengths, and compare the real and estimated values. The uniform distribution is denoted by $U(\bar{w}, l)$, where $\bar{w}$ represents the mean of the distributed commitment strengths, l represents the length of an interval between the minimum and maximum commitment strength.

Figure S5 shows the distances and the relative errors of *waning commitment* and *increasing commitment*, where the distance is calculated as $|p_c(U(\bar{w}, l)) - p_c(\bar{w})|$, while the relative error is calculated as $\frac{|p_c^{est}(U(\bar{w},l)) - p_c^{real}(U(\bar{w},l))|}{p_c^{real}(U(\bar{w},l))}$; $p_c(U(\bar{w}, l))$ represents the critical value of a system with commitment strengths $w \sim U(\bar{w}, l)$, the distance value of point ($\bar{w} = 10$, $l = 2$, est) is calculated as $|p_c^{est}(U(10, 2)) - p_c(10)|$. In both figures, the estimated distances are very close to the real ones,

$$\begin{aligned} |p_c^{est}(U(\bar{w}, 2)) - p_c(\bar{w})| \approx |p_c^{real}(U(\bar{w}, 2)) - p_c(\bar{w})| \\ |p_c^{est}(U(\bar{w}, 8)) - p_c(\bar{w})| \approx |p_c^{real}(U(\bar{w}, 8)) - p_c(\bar{w})| \end{aligned} \tag{S4}$$

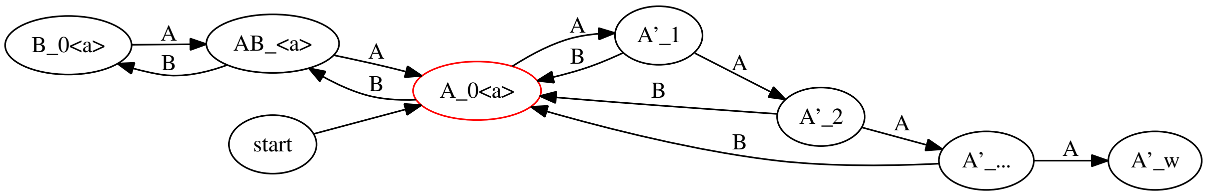

Figure S3: Substates transition graph of the *increasing commitment naming game*. $B_{0<a>}, AB_{<a>}, A_{0<a>}$, $A'_1, ..., A'_{w-1}$ are the substates of an agent trying to commit to opinion A. Specifically, $B_{0<a>}, AB_{<a>}, A_{0<a>}$ are the substates of an agent able to commit to opinion A, currently holding opinion B, AB, A. A'_i is a substate, where the last symbol represents this agent after consecutively receiving opinion A i times. This agent will become committed after continuing to consecutively receive opinion A another $w - i$ times.

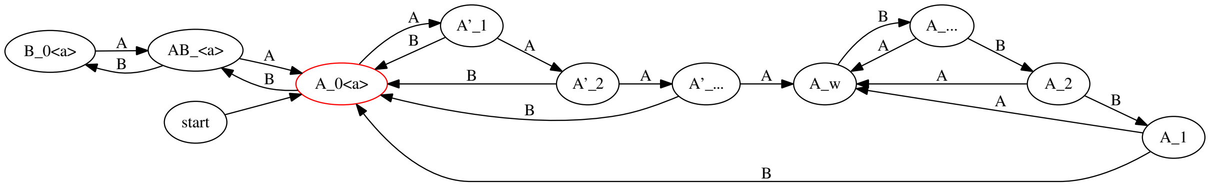

Figure S4: Substates transition graph of the *variable commitment naming game* where all the nodes with the ability to commit begin uncommitted. Initial conditions have very strong effects on the dynamics of the system. Since in our experiments, *variable commitment* is used to show the features of *increasing commitment*, using same initial conditions for *increasing commitment* and *variable commitment* allows for a close comparison of these models. By default, initial conditions were chosen in this natural way to fit the goals of each experiment.

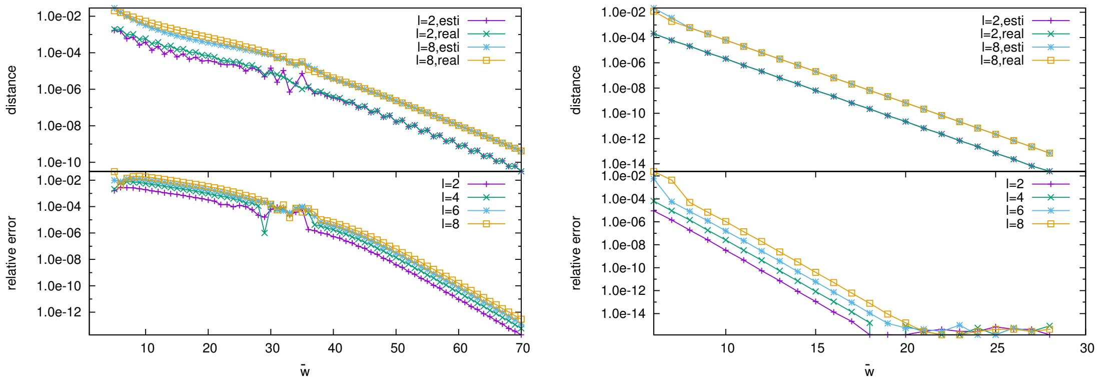

Figure S5: The distances and relative errors of critical values in the distributed *waning commitment naming game* (left plot), and *increasing commitment naming game* (right plot).

Before Interaction	Opinion	After Interaction	*Mean-Field*
$A- > A_{0<k>}, k \in \{n, b\}$	A	$A - A_{0<k>}$	
$A- > A_{0<a>}$	A	$A - A'_1$	$\frac{da_{0<a>}}{dt} = -a * a_{0<a>}, \frac{da'_1}{dt} = a * a_{0<a>}$
$A- > A'_i, i \in [1, w-1]$	A	$A - A'_{i+1}$	$\frac{da'_i}{dt} = -a * a'_i, \frac{da'_{i+1}}{dt} = a * a'_i$
$A- > A'_w$	A	$A - A'_w$	
$A- > B_{0<k>}, k \in \{n, a, b\}$	A	$A - AB_{<k>}$	$\frac{db_{0<k>}}{dt} = -a * b_{0<k>}, \frac{dc_{<k>}}{dt} = a * b_{0<k>}$
$A- > B'_{j'}, j' \in [1, w'-1]$	A	$A - B_{0<b>}$	$\frac{db'_{j'}}{dt} = -a * b'_{j'}, \frac{db_{0<b>}}{dt} = a * b'_{j'}$
$A- > B'_{w'}$	A	$A - B'_{w'}$	
$A- > AB_{<k>}, k \in \{n, a, b\}$	A	$A - A_{0<k>}$	$\frac{dc_{<k>}}{dt} = -a * c_{<k>}, \frac{da_{0<k>}}{dt} = a * c_{<k>}$
$B- > A_{0<k>}, k \in \{n, a, b\}$	B	$B - AB_{<k>}$	$\frac{da_{0<k>}}{dt} = -b * a_{0<k>}, \frac{dc_{<k>}}{dt} = b * a_{0<k>}$
$B- > A'_j, j \in [1, w-1]$	B	$B - A_{0<a>}$	$\frac{da'_j}{dt} = -b * a'_j, \frac{da_{0<a>}}{dt} = b * a'_j$
$B- > A'_w$	B	$B - A'_w$	
$B- > B_{0<k>}, k \in \{n, a\}$	B	$B - B_{0<k>}$	
$B- > B_{0<b>}$	B	$B - B'_1$	$\frac{db_{0<b>}}{dt} = -b * b_{0<b>}, \frac{db'_1}{dt} = b * b_{0<b>}$
$B- > B'_{i'}, i' \in [1, w'-1]$	B	$B - B'_{i'+1}$	$\frac{db'_{i'}}{dt} = -b * b'_{i'}, \frac{db'_{i'+1}}{dt} = b * b'_{i'}$
$B- > B_{w'}$	B	$B - B_{w'}$	
$B- > AB_{<k>}, k \in \{n, a, b\}$	B	$B - B_{0<k>}$	$\frac{dc_{<k>}}{dt} = -b * c_{<k>}, \frac{db_{0<k>}}{dt} = b * c_{<k>}$
$AB_{<k_1>}- > A_{0<k_2>}$, $k_1 \in \{n, a, b\}, k_2 \in \{n, b\}$	$A(50\%)$	$A_{0<k_1>} - A_{0<k_2>}$	$\frac{dc_{<k_1>}}{dt} = -c_{<k_1>} * a_{0<k_2>}/2, \frac{da_{0<k_1>}}{dt} = c_{<k_1>} * a_{0<k_2>}/2$
$AB_{<k>}- > A_{0<a>}, k \in \{n, a, b\}$	$A(50\%)$	$A_{0<k>} - A'_1$	$\frac{dc_{<k>}}{dt} = -c_{<k>} * a_{0<a>}/2, \frac{da_{0<k>}}{dt} = c_{<k>} * a_{0<a>}/2,$ $\frac{da_{0<a>}}{dt} = -c_{<k>} * a_{0<a>}/2, \frac{da'_1}{dt} = c_{<k>} * a_{0<a>}/2$
$AB_{<k>}- > A'_i, i \in [1, w-1], k \in \{n, a, b\}$	$A(50\%)$	$A_{0<k>} - A'_{i+1}$	$\frac{dc_{<k>}}{dt} = -c_{<k>} * a'_i/2, \frac{da_{0<k>}}{dt} = c_{<k>} * a'_i/2,$ $\frac{da'_i}{dt} = -c_{<k>} * a'_i/2, \frac{da'_{i+1}}{dt} = c_{<k>} * a'_i/2$
$AB_{<k>}- > A'_w, k \in \{n, a, b\}$	$A(50\%)$	$A_{0<k>} - A'_w$	$\frac{dc_{<k>}}{dt} = -c_{<k>} * a'_w/2, \frac{da_{0<k>}}{dt} = c_{<k>} * a'_w/2$
$AB- > B_{0<k>}, k \in \{n, a, b\}$	$A(50\%)$	$AB - AB_{<k>}$	$\frac{db_{0<k>}}{dt} = -c * b_{0<k>}/2, \frac{dc_{<k>}}{dt} = c * b_{0<k>}/2$
$AB- > B'_{j'}, j' \in [1, w'-1]$	$A(50\%)$	$AB - B_{0<b>}$	$\frac{db'_{j'}}{dt} = -c * b'_{j'}/2, \frac{db_{0<b>}}{dt} = c * b'_{j'}/2$
$AB- > B'_{w'}$	$A(50\%)$	$AB - B'_{w'}$	
$AB_{<k_1>}- > AB_{<k_2>}, k_1, k_2 \in \{n, a, b\}$	$A(50\%)$	$A_{0<k_1>} - A_{0<k_2>}$	$\frac{dc_{<k_1>}}{dt} = -c_{<k_1>} * c_{<k_2>}/2, \frac{dc_{<k_2>}}{dt} = -c_{<k_1>} * c_{<k_2>}/2,$ $\frac{da_{0<k_1>}}{dt} = c_{<k_1>} * c_{<k_2>}/2, \frac{da_{0<k_2>}}{dt} = c_{<k_1>} * c_{<k_2>}/2$
$AB- > A_{0<k>}, k \in \{n, a, b\}$	$B(50\%)$	$AB - AB_{<k>}$	$\frac{da_{0<k>}}{dt} = -c * a_{0<k>}/2, \frac{dc_{<k>}}{dt} = c * a_{0<k>}/2$
$AB- > A'_j, j \in [1, w-1]$	$B(50\%)$	$AB - A_{0<a>}$	$\frac{da'_j}{dt} = -c * a'_j/2, \frac{da_{0<a>}}{dt} = c * a'_j/2$
$AB- > A'_w$	$B(50\%)$	$AB - A'_w$	
$AB_{<k_1>}- > B_{0<k_2>}$, $k_1 \in \{n, a, b\}, k_2 \in \{n, a\}$	$B(50\%)$	$B_{0<k_1>} - B_{0<k_2>}$	$\frac{dc_{<k_1>}}{dt} = -c_{<k_1>} * b_{0<k_2>}/2, \frac{db_{0<k_1>}}{dt} = c_{<k_1>} * b_{0<k_2>}/2$
$AB_{<k>}- > B_{0<b>}, k \in \{n, a, b\}$	$B(50\%)$	$B_{0<k>} - B'_1$	$\frac{dc_{<k>}}{dt} = -c_{<k>} * b_{0<b>}/2, \frac{db_{0<k>}}{dt} = c_{<k>} * b_{0<b>}/2,$ $\frac{db_{0<b>}}{dt} = -c_{<k>} * b_{0<b>}/2, \frac{db'_1}{dt} = c_{<k>} * b_{0<b>}/2$
$AB_{<k>}- > B'_{i'}, i' \in [1, w'-1], k \in \{n, a, b\}$	$B(50\%)$	$B_{0<k>} - B'_{i'+1}$	$\frac{dc_{<k>}}{dt} = -c_{<k>} * b'_{i'}/2, \frac{db_{0<k>}}{dt} = c_{<k>} * b'_{i'}/2,$ $\frac{db'_{i'}}{dt} = -c_{<k>} * b'_{i'}/2, \frac{db'_{i'+1}}{dt} = c_{<k>} * b'_{i'}/2$
$AB_{<k>}- > B'_{w'}, k \in \{n, a, b\}$	$B(50\%)$	$B_{0<k>} - B'_{w'}$	$\frac{dc_{<k>}}{dt} = -c_{<k>} * b'_{w'}/2, \frac{db_{0<k>}}{dt} = c_{<k>} * b'_{w'}/2$
$AB_{<k_1>}- > AB_{<k_2>}, k_1, k_2 \in \{n, a, b\}$	$B(50\%)$	$B_{0<k_1>} - B_{0<k_2>}$	$\frac{dc_{<k_1>}}{dt} = -c_{<k_1>} * c_{<k_2>}/2, \frac{dc_{<k_2>}}{dt} = -c_{<k_1>} * c_{<k_2>}/2,$ $\frac{db_{0<k_1>}}{dt} = c_{<k_1>} * c_{<k_2>}/2, \frac{db_{0<k_2>}}{dt} = c_{<k_1>} * c_{<k_2>}/2$

Table S3: The list of all interactions of the *increasing commitment naming game*.

Derivatives	*Mean-Field* $r = b + c/2 = (a-1)(a-2)/2$
$da_{0<n>}/dt = 0$	$(3a/2 + c) * c_{<n>} - r * a_{0<n>} = 0$
$da_{0<b>}/dt = 0$	$(3a/2 + c) * c_{<b>} - r * a_{0<b>} = 0$
$da_{0<a>}/dt = 0$	$-a_{0<a>} + (3a/2 + c) * c_{<a>} + r * (a'_1 + ... + a'_{w-1}) = 0$
$da'_1/dt = 0$	$-a'_1 + (1-r) * a_{0<a>} = 0$
...	...
$da'_{w-1}/dt = 0$	$-a'_{w-1} + (1-r) * a'_{w-2} = 0$
$da'_w/dt = 0$	$(1-r) * a'_{w-1} = 0$
$db_{0<n>}/dt = 0$	$(3b/2 + c) * c_{<n>} - (1-r) * b_{0<n>} = 0$
$db_{0<a>}/dt = 0$	$(3b/2 + c) * c_{<a>} - (1-r) * b_{0<a>} = 0$
$db_{0<b>}/dt = 0$	$-b_{0<b>} + (3b/2 + c) * c_{<b>} + (1-r) * (b'_1 + ... + b'_{w'-1}) = 0$
$db'_1/dt = 0$	$-b'_1 + r * b_{0<b>} = 0$
...	...
$db'_{w'-1}/dt = 0$	$-b'_{w'-1} + r * b'_{w'-2} = 0$
$db'_{w'}/dt = 0$	$r * b'_{w'-1} = 0$
$dc_{<n>}/dt = 0$	$(1-r) * b_{0<n>} + r * a_{0<n>} - (3a/2 + 3b/2 + 2c) * c_{<n>} = 0$
$dc_{<a>}/dt = 0$	$(1-r) * b_{0<a>} + r * a_{0<a>} - (3a/2 + 3b/2 + 2c) * c_{<a>} = 0$
$dc_{<b>}/dt = 0$	$(1-r) * b_{0<b>} + r * a_{0<b>} - (3a/2 + 3b/2 + 2c) * c_{<b>} = 0$

Table S4: Overall derivatives of the *increasing commitment naming game*.

Besides, the relative errors are around 1% for small $\bar{w}$, and negligible for large $\bar{w}$.

3.2 Uniformly Distributed Commitment Strength

In the main paper, we analyze the critical values with general distribution of commitment strength. Here, we show a special case of uniform distribution analysis. We first compare the critical values of systems with commitment strengths $w \sim U(\bar{w}, 0)$ and $w \sim U(\bar{w}, 2), (\bar{w} > 1)$. As analyzed above, the exponential part of the critical value function is $kq^w, (0 < q < 1)$ for some constant k. According to Eq. S3,

$$\begin{aligned} p_c(U(\bar{w},0)) &= p_c(\bar{w}) = p_c(\infty) + kq^{\bar{w}} \\ p_c(U(\bar{w},2)) &= \frac{p_c(\bar{w}-1) + p_c(\bar{w}) + p_c(\bar{w}+1)}{3} = p_c(\infty) + k\frac{q^{\bar{w}-1} + q^{\bar{w}} + q^{\bar{w}+1}}{3} \end{aligned} \tag{S5}$$

$$\frac{q^{\bar{w}-1} + q^{\bar{w}} + q^{\bar{w}+1}}{3} - q^{\bar{w}} = \frac{q^{\bar{w}-1}(1-q)^2}{3} > 0 \tag{S6}$$

Thus, in the *waning commitment naming game* ($k > 0$), $p_c(U(\bar{w},2)) > p_c(U(\bar{w},0))$, while in the *increasing commitment naming game* ($k < 0$), $p_c(U(\bar{w},2)) < p_c(U(\bar{w},0))$.
Then, we compare the critical values of two general uniform distributions $w \sim U(\bar{w}, l)$ and $w \sim U(\bar{w}, l-2)$, $l \geq 2, \bar{w} > l/2$:

$$\begin{aligned} p_c(U(\bar{w},l)) &= p_c(\infty) + k\frac{q^{\bar{w}-\frac{l}{2}} + ... + q^{\bar{w}+\frac{l}{2}}}{l+1} \\ p_c(U(\bar{w},l-2)) &= p_c(\infty) + k\frac{q^{\bar{w}-\frac{l-2}{2}} + ... + q^{\bar{w}+\frac{l-2}{2}}}{l-1} \end{aligned} \tag{S7}$$

$$\frac{q^{\bar{w}-\frac{l-2}{2}} + ... + q^{\bar{w}+\frac{l-2}{2}}}{l-1} - \frac{q^{\bar{w}-\frac{l}{2}} + ... + q^{\bar{w}+\frac{l}{2}}}{l+1} = \frac{q^{\bar{w}-\frac{l}{2}}}{1-q}\left(\frac{q-q^l}{l-1} - \frac{1-q^{l+1}}{l+1}\right) \tag{S8}$$

$$\frac{\partial(\frac{q-q^l}{l-1}-\frac{1-q^{l+1}}{l+1})}{\partial q}=\frac{(1-q^{l-1})+...+(q^{l-1}-q^{l-1})}{l-1}(1-q)>0 \tag{S9}$$

When $q \to 1$, $\frac{q-q^l}{l-1}-\frac{1-q^{l+1}}{l+1} \to 0$, therefore when $0<q<1$, $\frac{q-q^l}{l-1}-\frac{1-q^{l+1}}{l+1}<0, \therefore \frac{q^{\bar{w}-\frac{l-2}{2}}+...+q^{\bar{w}+\frac{l-2}{2}}}{l-1} < \frac{q^{\bar{w}-\frac{l}{2}}+...+q^{\bar{w}+\frac{l}{2}}}{l+1}$. Thus, in the *waning commitment naming game* $(k>0)$, $p_c(U(\bar{w},l)) > p_c(U(\bar{w},l-2))$, while in the *increasing commitment naming game* $(k<0)$, $p_c(U(\bar{w},l)) < p_c(U(\bar{w},l-2))$. As we know, in a uniform distribution $U(\bar{w},l)$, standard deviation is $\sigma=\frac{l}{2\sqrt{3}}$. Therefore, the critical values change with the standard deviation.

3.3 Jensen's Inequality

According to Jensen's Inequality (*1*), if $f(\cdot)$ is a strictly convex function,

$$\frac{f(y)-f(x)}{y-x}<\frac{f(z)-f(y)}{z-y}<\frac{f(u)-f(z)}{u-z}, x<y<z<u \tag{S10}$$

If $w_{a_i}=w_{a_j}$, let $x=w_{a_i}-1$, $y=w_{a_i}=w_{a_j}$, $z=w_{a_j}+1$; if $w_{a_i}<w_{a_j}$, let $x=w_{a_i}-1$, $y=w_{a_i}$, $z=w_{a_j}$, $u=w_{a_j}+1$. For a strictly convex function $p_c(\cdot)$,

$$\begin{aligned}\frac{p_c(w_{a_i})-p_c(w_{a_i}-1)}{w_{a_i}-(w_{a_i}-1)} &< \frac{p_c(w_{a_j}+1)-p_c(w_{a_j})}{(w_{a_j}+1)-w_{a_j}}\\ p_c(w_{a_i})-p_c(w_{a_i}-1) &< p_c(w_{a_j}+1)-p_c(w_{a_j})\\ p_c^{before} &< p_c^{after}\end{aligned} \tag{S11}$$

Similarly, for a strictly concave function $p_c(\cdot)$,

$$\begin{aligned}p_c(w_{a_i})-p_c(w_{a_i}-1) &> p_c(w_{a_j}+1)-p_c(w_{a_j})\\ p_c^{before} &> p_c^{after}\end{aligned} \tag{S12}$$

4 Simulations

Figure S6 shows that network size have no impact on the critical value in both *waning* ($p_c=0.1075$) and *variable commitment* ($p_c=0.494$). However, network size does change the order parameters of system away from the critical value. Figure S7 shows that, for the uniform distribution with standard deviation $\sigma=\frac{l}{2\sqrt{3}}$, when the standard deviation increases, the critical values in the *waning commitment* model also increase while in the *increasing commitment* model the critical values decrease.

References

1. J. L. W. V. Jensen, *Acta Math.* **30**, 175 (1906).

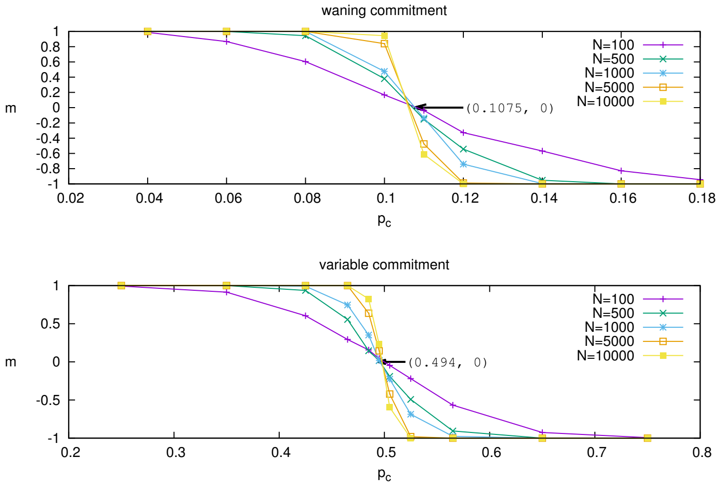

Figure S6: The order parameter values comparisons of single commitment group test of different network sizes from $N = 100$ to $N = 10000$. All the experiments are based on networks generated by Erdos-Renyi (ER) model with in which parameter $p \approx 0.01$, the average degree of each network is 10, and the commitment strengths w is 10. Each order parameter value is averaged over 500 runs. For each run, the order parameter value is either -1 or 1, which represents a system consensus on A or B.

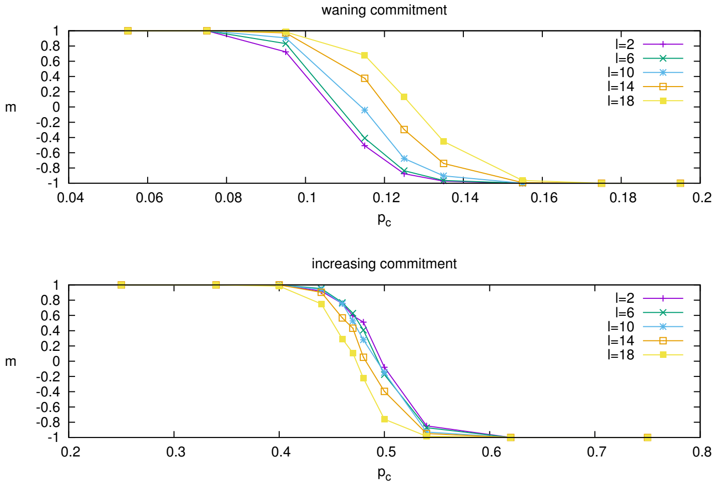

Figure S7: The order parameter values comparisons of single commitment group test of uniformly distributed commitment strength with different standard deviations. All the experiments are based on networks generated by Erdos-Renyi (ER) model with parameters $p \approx 0.01$, the average degree of each network is 10, and the mean of commitment strengths $\bar{w}$ of each distribution is 10. Each order parameter value is averaged over 500 runs. For each run, the order parameter value is either -1 or 1, which represents a system consensus on A or B.
